# Four-stage teaching technique and chest compression performance of medical students compared to conventional technique

**DOI:** 10.3325/cmj.2012.53.486

**Published:** 2012-10

**Authors:** Matej Jenko, Maja Frangež, Aleksander Manohin

**Affiliations:** Department of Anesthesiology, Faculty of Medicine, University of Ljubljana, Ljubljana, Slovenia

## Abstract

**Aim:**

To compare the 2-stage and 4-stage basic life support teaching technique. The second aim was to test if students’ self-evaluated knowledge was in accordance with their actual knowledge.

**Methods:**

A total of 126 first-year students of the Faculty of Medicine in Ljubljana were involved in this parallel study conducted in the academic year 2009/2010. They were divided into ten groups. Five groups were taught the 2-stage model and five the 4-stage model. The students were tested in a scenario immediately after the course. Questionnaires were filled in before and after the course. We assessed the absolute values of the chest compression variables and the proportions of students whose performance was evaluated as correct according to our criteria. The results were analyzed with independent samples *t* test or Mann-Whitney-U test. Proportions were compared with χ^2^ test. The correlation was calculated with the Pearson coefficient.

**Results:**

There was no difference between the 2-stage (2S) and the 4-stage approach (4S) in the compression rate (126 ± 13 min^-1^ vs 124 ± 16 min^ -1^, *P* = 0.180, independent samples *t* test), compression depth (43 ± 7 mm vs 44 ± 8 mm, *P* = 0.368, independent samples *t* test), and the number of compressions with correct hand placement (79 ± 32% vs 78 ± 12, *P* = 0.765, Mann-Whitney U-test). However, students from the 4-stage group had a significantly higher average number of compressions per minute (70 ± 13 min^ -1^ 2S, 78 ± 12 min^-1^ 4S, *P* = 0.02, independent samples *t* test). The percentage of students with all the variables correct was the same (13% 2S, 15% 4S, *P* = 0.741, χ^2^ test). There was no correlation between the students’ actual and self-evaluated knowledge (*P* = 0.158, Pearson coefficient = 0.127).

**Conclusions:**

The 4-stage teaching technique does not significantly improve the quality of chest compressions. The students’ self-evaluation of their performance after the course was too high.

Out-of-hospital cardiac arrest (OHCA) is a cessation of cardiac mechanical activity that can be recognized by the absence of signs of circulation in out-of-hospital setting (1). In most cases, it has a cardiac etiology and represents a significant health issue in many countries due to high mortality from cardiovascular diseases (2,3). Survival rates of OHCA remain poor, varying between 1% and 25% (4-7). Bystanders are present in more than 70% of cases (8) and bystander cardiopulmonary resuscitation (CPR) is a very important contributing factor in the survival of the victims (9-11). In cases when OHCA is a consequence of ventricular fibrillation (VF), the chances of survival decrease by 7%-10% per minute without CPR and by 3%-4% with proper CPR (12-16).

Several studies reported poor CPR quality, not only among laypeople, but also among health care professionals (17,18). A reason for this could be the quality of CPR courses, since both skill acquisition and retention have been shown to be poor after conventional training in CPR for laypeople (19-21). This may be caused by instructors’ overestimating the correct performance and the students’ believing their performance to be very good, despite poor results on the computerized manikin-based simulation (21,22).

New models of teaching were implemented at the University of Ljubljana in order to improve the results. One of them is the 4-stage approach, proposed by R. Peyton (Royal College of Surgeons) in 1998 (23-25). In a conventional, 2-stage approach, the instructors demonstrate the skill at slow speed and provide a commentary. This is followed by the student’s performing the skill under supervision. The model was criticized due to inadequate skills acquisition and retention. The 4-stage approach breaks down the skills teaching process into 4 stages: demonstration, deconstruction, formulation, and performance. The teachers first show the skill at a normal speed without commentary. Then they demonstrate the skill by breaking it into simple steps and add a commentary. In the third phase, they demonstrate it while being “guided through” the steps by the student. In the final phase, the students demonstrate and comment on the skill procedure.

Our study had two main aims. The first was to evaluate and compare the quality of the 2-stage and 4-stage teaching technique of the CPR course, using the quality of chest compression as the main criterion. The second was to compare the actual students’ performance with their self-evaluation in both teaching techniques.

## Methods

### Study characteristics

The study with parallel groups was conducted at the Simulation Center of the Faculty of Medicine, University of Ljubljana in the academic year 2009/2010. Ethical approval was received from the National Medical Ethics Committee of the Republic of Slovenia.

### The investigated skills

Although CPR still consists of chest compressions and ventilations, the role of the ventilations is disputable (26-28). The quality of chest compression is the major determinant of successful resuscitation (11). Parameters that determine the quality of chest compression can be measured objectively by the computerized manikin and compared to the instructor’s and trainees’ subjective assessment.

### Participants

The study included 126 first-year medical students. In order to make the teaching groups small, students were randomly assigned by random number generator (Microsoft Excel, Microsoft, Redmond, WA, USA) to 10 groups that participated in CPR classes at separate occasions during the academic semester. Five groups were taught the 4-stage teaching techniques (62 students) and five groups the 2-stage models (64 students). Each group consisted of 12 or 13 participants. Instructors were randomly assigned to a group also by random number generator.

The instructor had chosen a teaching technique before he was assigned to a group. Five instructors, skilled anesthesiologists who had received training in university didactics (29), were included in the study; two of them used the 4-stage and three of them the 2-stage approach. Most of them were also European Resuscitation Council (ERC) instructors. We presumed that the quality of the instructors was equal, it was constantly evaluated, and they were confident in both teaching techniques.

### Study design

The course for all the groups took place at the same location with the same equipment. Before the beginning of the course, the students filled in the first questionnaire. The duration of the CPR and automated external defibrillator (AED) course was four and a half hours. One of the study authors was present during the course, but was not actively involved in the course. After the course, students filled in the second questionnaire.

The students entered the room individually and received the same instructions: “The person you see suddenly collapsed. You are alone at the scene. We expect appropriate action from you. The manikin that represents the patient is prepared for the demonstration.”

Variables of chest compressions were recorded on the manikin. The demonstration ended two minutes after the beginning of the chest compression performance. During the course the students practiced on the same manikin that they later used in the demonstration (Resusci Anne Skill Reporter, Laerdal Medical, Stavanger, Norway).

The purpose of the study was explained to the students before the beginning of the course and they could choose not to participate, which two of them did.

### The questionnaire

The students self-evaluated the quality of their chest compressions before and after the course with grades from 1 (not competent to perform chest compression) to 4 (very competent to perform chest compressions with correct compression depth, rate, and hand placement) (supplementary questionnaire(web extra material 1)) (30). A similar questionnaire, based on the literature, was already used in a survey of CPR quality in 2005 (31).

### Observed variables of chest compressions

Variables of chest compressions were evaluated in accordance with the ERC guidelines 2005 and the Guidelines for Uniform Reporting of Measured Quality of Cardiopulmonary Resuscitation (32,33). We compared the absolute values of the observed variables between the 2-stage and the 4-stage approach. We also established the criteria how to evaluate each of the chest compressions variables (chest compression rate, chest compressions depth, and hand placement) as correct. Based on these criteria, we calculated the number of students with each correct variable and compared the number between both techniques. The observation of the variables was based on the printouts from the manikin.

### Chest compression depth

We calculated the average chest compression depth in the observed 2 minutes of chest compression performance. The variable was considered correct if it was within the range of 38 to 51 mm. We also set the condition that the number of too shallow compressions should not exceed 1/3 of all compressions in succession. A long sequence of shallow chest compression reduces chest compression effectiveness even if the average chest compression depth is within the correct range (34,35).

### Compressions rate

The variable was considered correct if the average rate was between 90 and 120 compressions per minute. The number of actual performed chest compressions per minute was also observed.

### Hand placement during chest compressions

The proportion of chest compressions with the correct hand placement was detected by manikin’s sensors. We decided to evaluate as correct those hand placements that were appropriate in 80% of cases or more.

**Figure 1:**
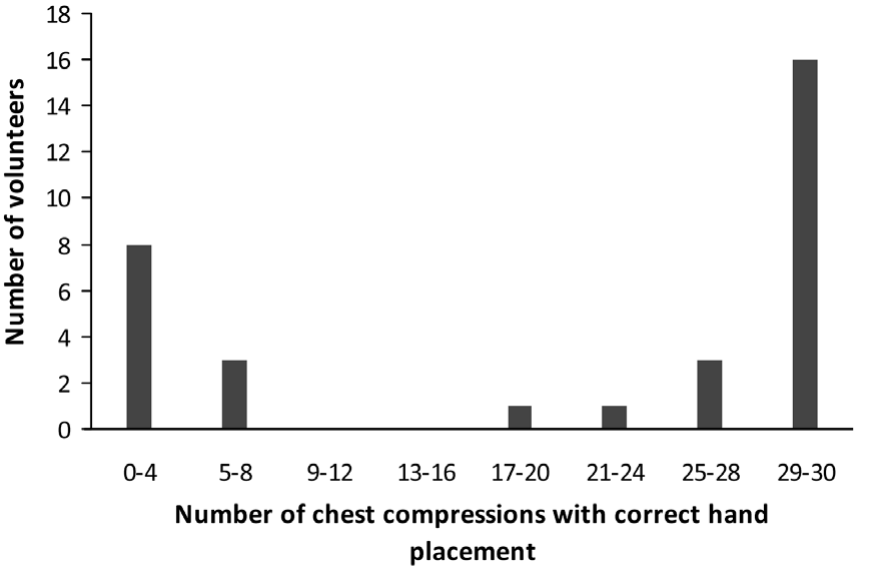
Chart from a study (38) that served as basis for our criteria. In the chart, the x-axis indicates the number of correctly placed compressions and the y-axis indicates the number of volunteers achieving this number.

Our review of the literature showed that the students achieved the result of either below 10% or above 80%, which in practice is displayed as a U shaped distribution (36-38). Our criterion was based on another study (38), as is shown in Figure 1. It shows that it is realistic and practically feasible to achieve the result of 25 out of 30 correct chest compressions per cycle (80%). A similar criterion (76% of correct chest compression) was used in another study (39).

### Statistical analysis

*Power of the study* Sample size calculation was done on the basis of other studies (17,26,30,40). We proposed 55% chest compressions with a correct depth in 2-stage teaching technique and 80% in 4-stage teaching technique group. Using χ^2^, we determined that 126 participants (64 in 4-stage and 62 in 2-stage) would result in power of the study of 85% (double sided α = 0.05).

Eighty percent or more chest compressions with correct depth would mean significant improvement compared to other models of teaching.

*Statistical tests.* Data were analyzed using SPSS 17.0 (SPSS Inc., Chicago, IL, USA). Normally distributed data were analyzed using two independent samples *t* test and other data were analyzed using Mann-Whitney U-test. Students with correct variables were compared with χ^2^ or Fisher exact test where appropriate. The questionnaires were analyzed using Mann-Whitney U-test. The correlation between actual performance and self-evaluation was calculated using the Pearson correlation coefficient. *P* < 0.05 was considered statistically significant.

*Difference between different groups in the same teaching technique*. Chest compressions rate, chest compressions depth, and hand placement during chest compressions were compared between the all 5 groups of same teaching technique using the Kruskal-Wallis test.

## Results

There was no difference between the 2-stage and the 4-stage approach in the compression rate (126 ± 13 min^-1^ vs 124 ± 16 min^ -1^, *P* = 0.180, independent samples *t* test), compression depth (43 ± 7 mm vs 44 ± 8 mm, *P* = 0.368, independent samples *t* test), and the number of compressions with correct hand placement (79 ± 32% vs 78 ± 12, *P* = 0.765, Mann-Whitney U-test). However, students from the 4-stage group had a significantly higher average number of compressions per minute (70 ± 13 min^-1^ 2S, 78 ± 12 min^ -1^ 4S, *P* = 0.02, independent samples *t* test). The percentage of students with all the variables correct was the same (13% in two-stage and 15% in four-stage approach, *P* = 0.741, χ^2^ test). (Table 1). Relative students’ success did not differ significantly between the two models of teaching (Table 2).

**Table 1:** Comparison of chest-compression variables in both teaching techniques

Chest compression variable	4-stage teaching technique	2-stage teaching technique	*P* value
Chest compressions rate* (min^-1^)	124 ± 16 122 (114-138)^‡^	126 ± 13 126 (118-137)^‡^	0.180
performed chest compressions* (min^-1^)	78 ± 12 78 (71-88)	70 ± 13 71 (60-80)	0.020^║^
Average compression depth* (mm)	44 ± 8 45 (39-51)	43 ± 7 44 (37-48)	0.368
Percentage of chest compressions with correct depth^†^ (%)	52 ± 33 56 (20-82)	59 ± 32 71 (36-85)	0.248
Percentage of chest compressions >51mm^†^ (%)	26 ± 33 9 (0-52)	17 ± 28 3 (0-21)	0.143
Percentage of chest compressions <38 mm^†^ (%)	21 ± 32 3 (0-36)	23 ± 31 5 (0-44)	0.538
Percentage of chest compression with correct hand placement^†^ (%)	78 ± 32 100 (62-100)	79 ± 32 100 (58-100)	0.765
Proportion of chest compression with incomplete release^†^ (%)	5 (0-8) 0 (0-1)	2 ± 9 0 (0-0)	0.090

*Data were analyzed using two independent samples *t* test.

†Data were analyzed using Mann-Whitney U test.

‡First row within each cell are averages and standard errors, while the second row are medians and 25th and 75th percentiles.

§Statistically significant difference.

**Table 2:** Comparison of relative students’ success in both models of teaching

	Number (%) of students		
Chest compression variable	4-stage approach (N = 62)	2-stage approach (N = 64)	*P* value
Correct chest compression depth	28 (45)	35 (55)	0.138
Correct chest compression rate	26 (42)	17 (26)	0.147*
Chest compression rate >120 min^-1^	34 (55)	47 (74)	0.090*
Chest compression rate <90min^-1^	2 (3)	0	0.148^†^
Correct hand placement	40 (65)	43 (67)	0.752*
Correct depth, rate, and hand placement for the same student	9 (15)	8 (13)	0.741*

*χ^2^ test.

†Fisher exact test.

**Figure 2:**
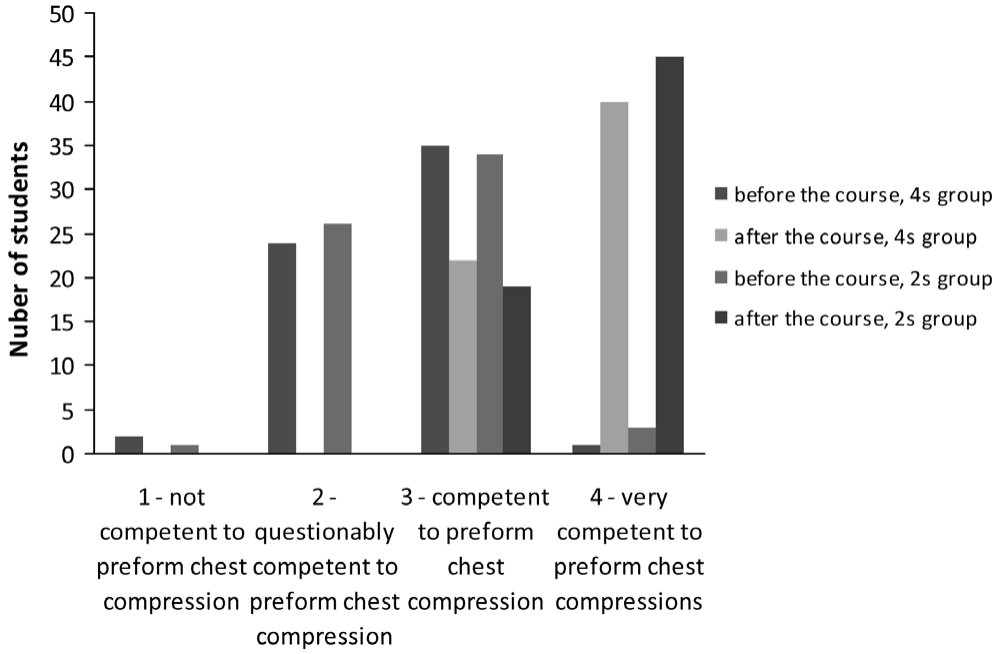
Self-evaluation of students’ knowledge before and after the course.

The average grade in the students’ self-evaluation before the course was 2.6 ± 0.6 in both teaching techniques (*P* = 0.805, Mann-Whitney test U). The average grade after the course was 3.7 ± 0.47 in 2-stage approach and 3.6 ± 0.48 in 4-stage approach, with no significant difference between the groups (*P* = 0.616, Mann-Whitney test U) (Figure 2).

**Figure 3:**
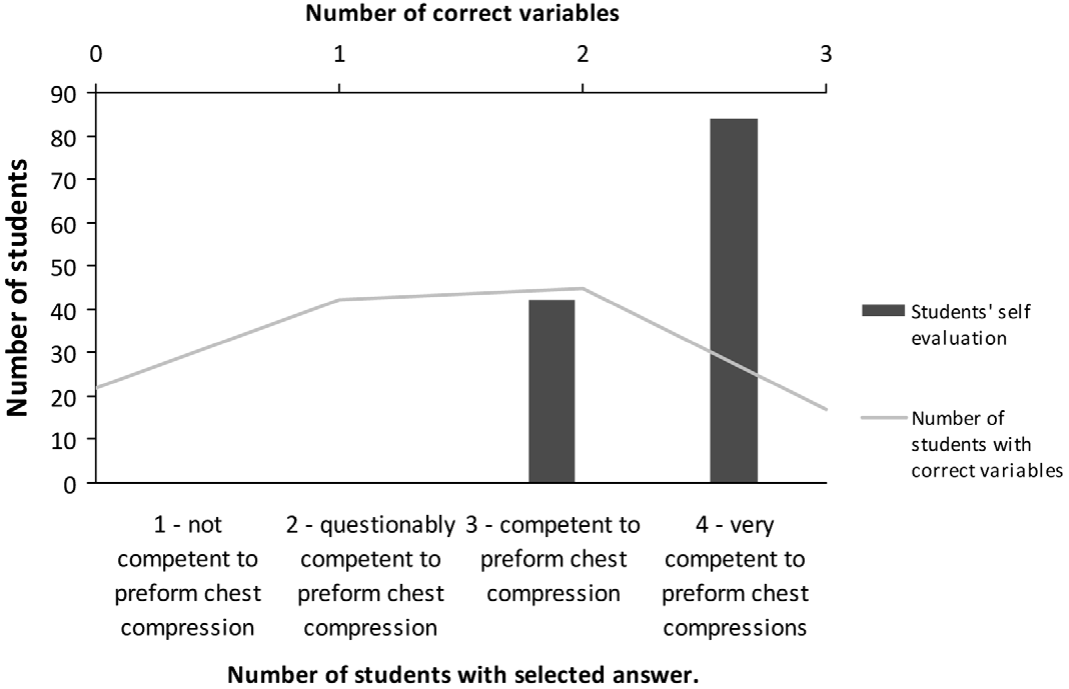
Comparison of the self-evaluation and number of correct variables. 0 means that none of the observed variables was correct; 1 means that any one of the following variables was correct: chest compression depth, chest compression rate, or hand placement; 2 means that any two of those variables were correct; and 3 that all of them were correct.

There was no significant correlation between the self-evaluation of skill performance and the actual number of correct variables (*P* = 0.158 Pearson correlation coefficient = 0.127) (Figure 3).

There were no significant differences between different groups using the same teaching technique (groups using 2-stage technique: *P* = 0.114 for chest compression depth, *P* = 0.310 for chest compression rate, *P* = 0.410 for correct hand placement; 4-stage technique: *P* = 0.720 for chest compression depth, *P* = 0.126 for chest compression rate, *P* = 0.100 for correct hand placement, Kruskal-Wallis test)

## Discussion

In our study, we did not find any difference between the 4-stage and 2-stage teaching technique in chest compressions rate, depth of hand placement, or incomplete release. There was, however, a significant difference in the number of compressions per minute. The proportion of students who could perform chest compressions correctly was the same in both teaching techniques, but it was low. There was no correlation between the students’ self-evaluation and their actual skill performance.

### Average chest compression rate

The chest compressions rate was higher than recommended in the guidelines and was not affected by the teaching technique. Other studies presented inconclusive results. A study that included three hospitals in the USA reported a too low rate and showed that the survival was higher if the rate was over 90 compressions per minute (41). A study conducted in Finland had comparable time of observation with a previous study (eight and ten minutes), but the chest compression rate was above the recommended value (42). Our time of observation was shorter, but our results are similar to those in Finland. A too high chest compressions rate (over 120 compressions per minute) can decrease the survival rate (43).

It seems that even the 4-stage technique does not provide accurate feedback to the trainees about the rate of their chest compressions performance. Most of students evaluated their chest compressions rate as correct (Figure 3). In order to improve this variable, a metronome should be used independently of the teaching technique, as it was shown to significantly improve the ability of students to produce a chest compression rate close to the ideal (42,44-46).

### Average chest compression depth

The number of chest compressions with correct depth was unsatisfactory in both teaching techniques. The number of students with this variable evaluated as correct and the number of chest compressions with correct depth was lower than in other studies (27,30,41,42,47). The absolute value of chest compression depth was similar to other comparable studies. We conclude that the poorer results in our study are the consequence of the additional condition that no more than 1/3 of chest compression should be less than 38 mm deep. Changes in the ERC 2010 guidelines point out the fact that shallow compressions significantly decrease survival and their number should be minimized (35). Both 2-stage and 4-stage teaching techniques should be improved with automated feedback device, which would provide real-time information to the trainee. Some studies with that improvement report 80% of correct chest compressions (40,48-51).

### Correct hand placement

There was no difference in the proportion of chest compressions with correct hand placement between both teaching techniques. The 4-stage teaching technique might show its advantage if the instructions how to find the correct hand position were more complex. There was a greater number of incorrect hand placements in trainings using the less complex 2005 guidelines for the 4-stage technique than in the trainings using 2000 guidelines (52,53).

The studies that used the manikin printout for evaluation of hand placement showed a similar number of correct chest compressions as our study (38,54,55). However, the number of correct chest compressions in the study with only visual assessment was significantly higher (53).

### Difference in the number of performed chest compressions per minute

The variable that differed significantly between the teaching techniques was the number of performed chest compressions per minute. This variable was related to the chest compression rate and to the pauses between chest compressions for ventilation, defibrillation, and other reasons.

The chest compression rate was roughly the same in both teaching techniques, which suggests that 2-stage technique group had longer pauses between the chest compression intervals. The only other procedure performed was mouth to mouth ventilations. It was shown that pauses during chest compressions significantly decreased the chances of survival (56-58).

The literature suggests a link between the expertise of the trainees and the number of performed chest compressions per minute. In studies with lay people, the number was as low as 40 compressions per minute, while in studies with health professionals it was 80 (42,59,60).

### Difference between self-evaluation and actual knowledge

The self-evaluation of the students before the course was roughly the same in both groups. After the course, the students were very confident about their knowledge. However, if we compare self-evaluation to the actual results, self-confidence does not seem to be justified. All students who participated in the study had passed the CPR course before. In the instructor’s opinion, the variables of chest compression were performed well enough to provide effective chest compressions. But according to our criteria, only a minority of students performed well regarding chest compressions rate, depth, and correct hand placement.

The criteria that we used to evaluate the chest compressions of each individual student as correct were based on the ERC guidelines. It is not likely that the small number of students with correct chest compressions was a consequence of wrongly chosen criteria. During the course, no electronic feedback device was used; it was up to the instructor to decide whether the chest compressions were correct. As shown here, this decision is unreliable, even with an experienced instructor. As a consequence, the students received inaccurate feedback about their knowledge during the course. This was the primary reason for the difference between the actual knowledge and the students’ self evaluation.

### Comparison of both teaching techniques

There was no significant difference in most chest compression variables between the two teaching techniques. From the results of our and other studies, we can conclude that for good quality chest compressions it is not important how the skill is explained, but the duration of the actual skill performance on the manikin with appropriate real-time feedback. We believe that the time every trainee spends performing chest compressions (the hands-on time) is significantly more correlated with the chest compression quality than the instructor or teaching method (19,21,61-64).

There was, however, a significant difference in the number of performed chest compressions between the two teaching methods. This could be a way to improve the survival rates with a different teaching technique if the quality of the ventilations is the same in both teaching techniques. No advantages of the 4-stage teaching technique have been reported, even in teaching complex skills (23,65). We do not have any evidence that the ventilation was better in the 4-stage teaching technique. The reason for the shorter time may be the combination of a faster performance of all the steps of the ventilation (lifting the chin, starting to ventilate) and faster finding of the correct hand placement and starting the chest compressions earlier.

### Study limitations

The sample size was quite large compared to similar studies, but more than one instructor was included in each teaching technique. We showed that there were no significant differences in the observed chest compression variables among different groups using the same teaching technique. Consequently, there were no significant differences among the instructors of the same teaching technique, but it is possible, although not likely, that the instructors of the two techniques had significantly different quality.

The population observed in this study were medical students. They were at the beginning of their first year and their knowledge was not very different from the laypersons’ but their motivation was probably higher, so the results of the questionnaire are not completely applicable to the laymen CPR courses (66).

In conclusion, the 4-stage teaching technique does not significantly improve the quality of the chest compressions. Changes in CPR course**s** are needed in order to improve chest-compression quality, and to provide trainees with more accurate feedback.

## Additional Material

Supplementary questionnaire
