# Supplementary material for: Four-stage teaching technique and chest compression performance of medical students compared to conventional technique
Source: Croat Med J. 2012 Oct;53(5):486–95. doi: 10.3325/cmj.2012.53.486 (PMC3490459; doi:10.3325/cmj.2012.53.486)
Supplement: Supplementary questionnaire [file CroatMedJ_53_s016.pdf]

Dear Student!

We would like to welcome you at the CPR/AED course.

Please answer the following question BEFORE the beginning of the course.

How confident do you feel about performing chest compressions during resuscitation?

1 - Not competent to perform chest compression.
2 - Questionably competent to perform chest compression.
3 - Competent to perform chest compression.
4 - Very competent to perform chest compressions.

We would like to thank you for attending CPR/AED course!

Please answer that question after the course.

How confident do you feel now about performing chest compressions during resuscitation?

1 - Not competent to perform chest compression.
2 - Questionably competent to perform chest compression.
3 - Competent to perform chest compression.
4 - Very competent to perform chest compressions.
